# Post-Traumatic Stress and Substance Use in Migrant Populations: A Literature Review

**DOI:** 10.1192/j.eurpsy.2026.10902

**Published:** 2026-07-31

**Authors:** A. R. Bianchi, G. Tedesco, L. Cattani, P. Brambilla, L. Del Fabro

**Affiliations:** 1 Department of Pathophysiology and Transplantation, University of Milan; 2 Department of Neurosciences and Mental Health, Fondazione IRCCS Ca’ Granda Ospedale Maggiore Policlinico, milan, Italy

## Abstract

**Introduction:**

Migrants and refugees are frequently exposed to traumatic experiences, resulting in high prevalence of post-traumatic stress disorder (PTSD). PTSD and substance use disorders (SUD) commonly co-occur, creating a challenging clinical presentation with important treatment implications. While this association is well established in the general population, less is known in migrants.

**Objectives:**

This literature review examined the association between post-traumatic symptoms and substance use among migrant individuals, aiming to clarify whether migration-related experiences confer specific risks for SUD.

**Methods:**

A systematic literature search was conducted in PubMed, Web of Science, and Scopus, covering publications from 2005 to 2025. The association between substance use and post-traumatic symptoms in migrant populations was examined in peer-reviewed observational studies.

**Results:**

While findings were mixed, the results largely suggest a significant association between PTSD and substance use in migrants. In studies comparing migrants and non-migrants, PTSD was consistently linked to an increased risk of SUD in both groups. The association appeared weaker in migrants overall, but when SUD was present, post-traumatic symptoms tended to be more severe than in non-migrants. Cross-sectional studies showed heterogeneous associations between post-traumatic symptoms and substance use. Moreover, some studies reported an association between the severity of post-traumatic symptoms and substance use in migrants.

**Conclusions:**

Preliminary evidence suggests that PTSD and substance use disorders co-occur in migrant populations, although the strength of this association varies across study designs, populations, and substances. Interpretation is limited by small sample sizes, the reliance on cross-sectional designs, and substantial heterogeneity across studies. Further research is needed to clarify the mechanisms linking PTSD and SUD in migrant populations, with particular attention to improved control of confounders and the use of more homogeneous samples.

**Disclosure of Interest:**

None Declared

